# Inhibition of p38 mitogen-activated protein kinase enhances c-Jun N-terminal kinase activity: Implication in inducible nitric oxide synthase expression

**DOI:** 10.1186/1471-2210-6-5

**Published:** 2006-02-21

**Authors:** Aleksi Lahti, Outi Sareila, Hannu Kankaanranta, Eeva Moilanen

**Affiliations:** 1 The Immunopharmacology Research Group, Medical School, University of Tampere and Tampere University Hospital, Tampere, Finland

## Abstract

**Background:**

Nitric oxide (NO) is an inflammatory mediator, which acts as a cytotoxic agent and modulates immune responses and inflammation. p38 mitogen-activated protein kinase (MAPK) signal transduction pathway is    activated by chemical and physical stress and regulates immune responses. Previous studies have shown that p38 MAPK pathway regulates NO production induced by inflammatory stimuli. The aim of the present study was to investigate the mechanisms involved in the regulation of inducible NO synthesis by p38 MAPK pathway.

**Results:**

p38 MAPK inhibitors SB203580 and SB220025 stimulated lipopolysaccharide (LPS)-induced inducible nitric oxide synthase (iNOS) expression and NO production in J774.2 murine macrophages. Increased iNOS mRNA expression was associated with reduced degradation of iNOS mRNA. Treatment with SB220025 increased also LPS-induced c-Jun N-terminal kinase (JNK) activity. Interestingly, JNK inhibitor SP600125 reversed the effect of SB220025 on LPS-induced iNOS mRNA expression and NO production.

**Conclusion:**

The results suggest that inhibition of p38 MAPK by SB220025 results in increased JNK activity, which leads to stabilisation of iNOS mRNA, to enhanced iNOS expression and to increased NO production.

## Background

Nitric oxide (NO) is a highly reactive signaling molecule and inflammatory mediator, which acts as a cytotoxic agent and modulates immune responses and inflammation [1,2]. High amounts of NO are produced for prolonged times by inducible nitric oxide synthase (iNOS) in response to proinflammatory cytokines and bacterial products [3,4]. iNOS expression is regulated both at transcriptional and posttranscriptional level. Several transcription factors which regulate iNOS promoter activity have been characterized, but the mechanisms and factors regulating iNOS mRNA stability are largely unknown [2,5].

Mitogen-activated protein kinases (MAPKs) are a family of serine/threonine kinases that are part of the signal transduction pathways, which connect inflammatory and various other extracellular signals to intracellular responses e.g. gene expression [6]. p38 MAPK and c-Jun N-terminal kinase (JNK) are members of the MAPK family, and they are activated by chemical and physical stress. p38 and JNK regulate immune responses and expression of various cytokines e.g. tumor necrosis factor-α, interleukin-1 and interleukin-6 [7].

JNK and p38 MAPK are also involved in regulation of iNOS expression. Previous studies have shown that JNK pathway belongs to the factors that mediate the up-regulation of iNOS expression [8-10]. Depending on the cell-type and stimulation used, p38 MAPK has been reported to have either up-regulatory role [11-13], down-regulatory role [14-16] or no role [17,18] in iNOS expression. We have previously reported that p38 MAPK inhibitors enhance iNOS expression and NO production in LPS-stimulated J774 macrophages [19]. The detailed mechanism behind those stimulatory effects is not known.

The aim of the present study was to investigate the mechanism by which p38 inhibition leads to increase in NO production. The results suggest that inhibition of p38 MAPK increases LPS-induced JNK activity, which leads to stabilisation of iNOS mRNA and increased production of NO in activated macrophages.

## Results

### p38 MAPK inhibitor SB220025 increases LPS-induced NO production and iNOS expression

**Figure 1:**
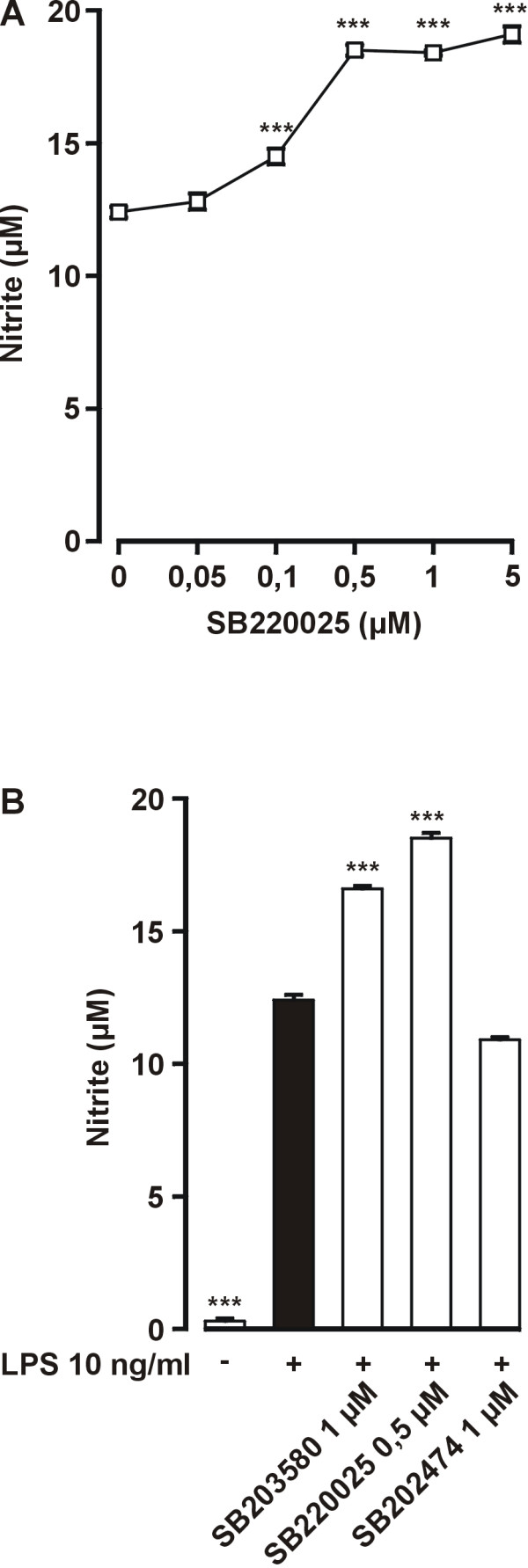
**Effect of p38 MAPK inhibitor SB220025 on LPS-induced NO production**. (A) Cells were stimulated with LPS (10 ng/ml) and treated with various concentrations of SB220025 1 h after LPS stimulation. After 24 h incubation the nitrite concentrations were measured as a marker of NO production. Values are mean ± S.E.M. (n = 6). (B) Cells were stimulated with LPS 1 h before addition of tested compounds. 24 h after addition of LPS the nitrite concentrations were measured as marker of NO production. Values are mean ± S.E.M. (n = 6). ***P < 0.001 compared with cells treated with LPS only.

**Figure 2:**
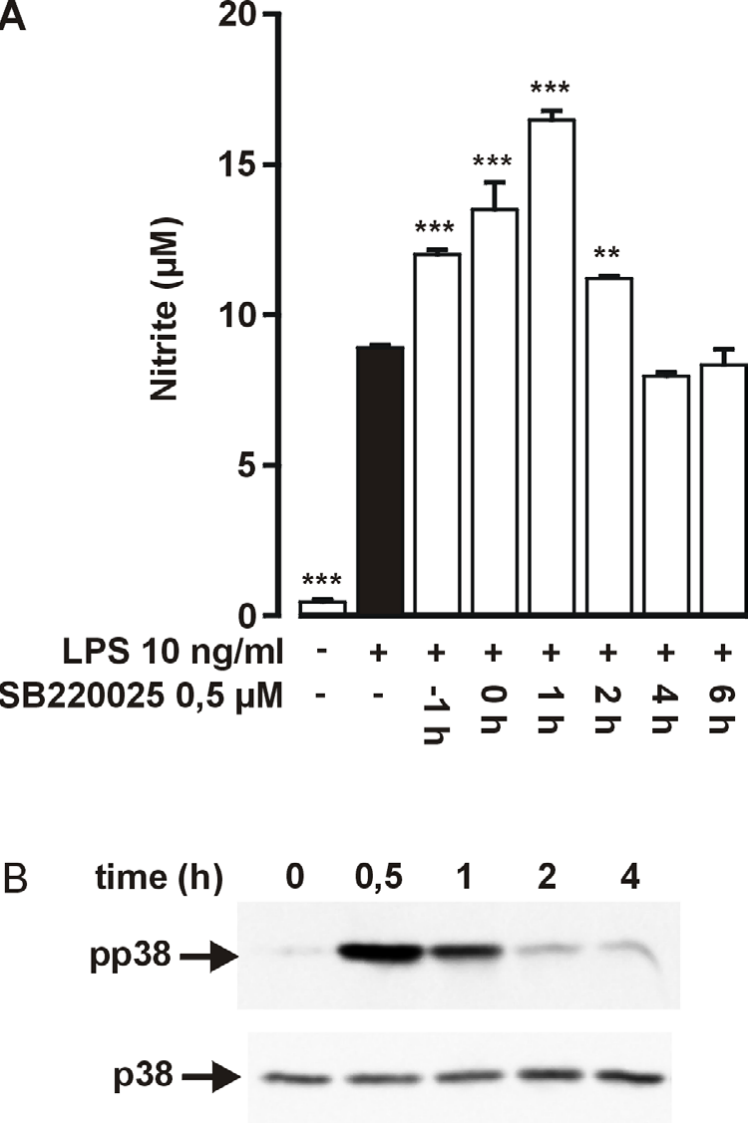
**The effect of SB220025 on NO production when added before or at various time points after LPS (A) and time course of p38 MAPK activation following LPS (B)**. (A) Cells were treated with SB220025 1 h before or at indicated time points after stimulation with LPS. After 24 h incubation, the nitrite concentrations were measured as a marker of NO production. Values are mean ± S.E.M. (n = 6). **P < 0.01, ***P < 0.001 compared with cells treated with LPS only. (B) Cells were stimulated with LPS (10 ng/ml) and incubations were terminated at indicated time points. Parallel immunoblots were run from same cell lysates using antibodies against phosphorylated p38 (pp38) and total p38. The result is representative of three experiments with similar results.

**Figure 3:**
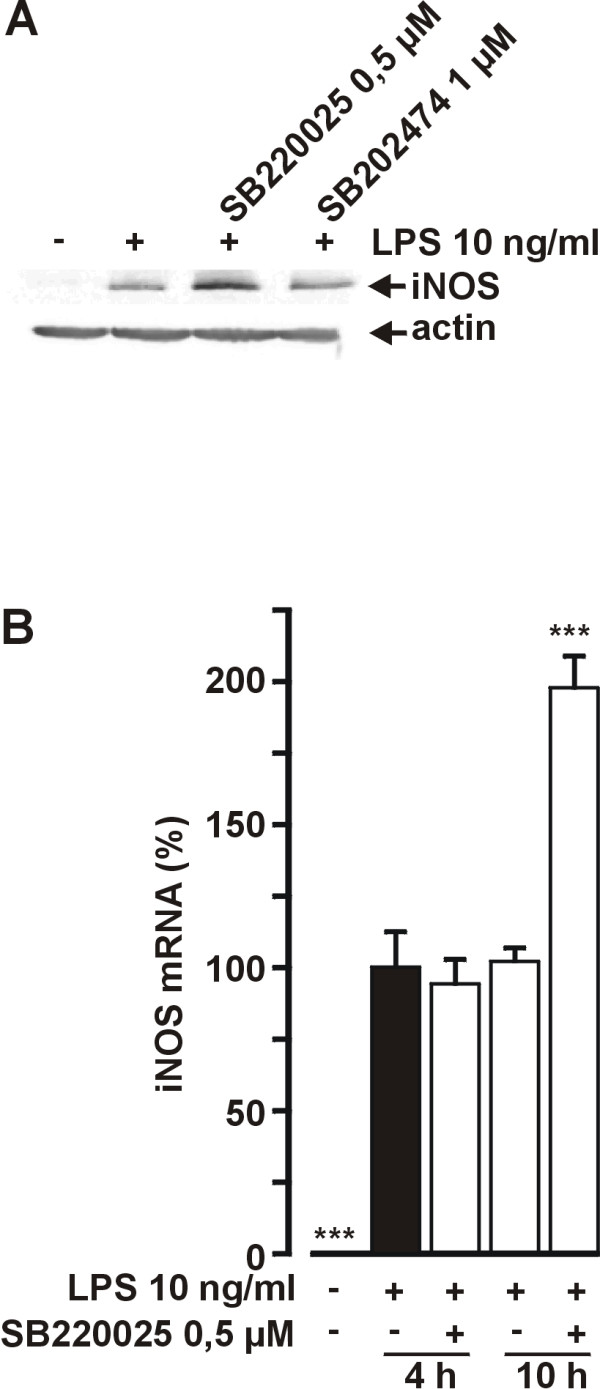
**Effect of p38 MAPK inhibitor SB220025 on LPS-induced iNOS expression**. (A) Cells were stimulated with LPS 1 h before addition of SB220025. After 24 h, incubations were terminated and immunoblots were run using antibody against iNOS and actin. The result is representative of three experiments with similar results. (B) Cells were stimulated with LPS 1 h before addition of SB220025. Incubations were terminated at indicated time points and extracted total RNA was subjected to real time RT-PCR. iNOS mRNA levels were normalised against GAPDH. Values are mean ± S.E.M. (n = 3). *P < 0.05, ***P < 0.001 compared with cells treated with LPS only.

We have previously shown that pyridinyl imidazole inhibitor of p38 MAPK SB203580 [20] stimulates LPS-induced NO production [19]. SB220025 is a recently developed potent and specific inhibitor of p38 MAPK with an IC_50 _value of 60 nM in kinase activity assay [21]. Figure 1A shows that SB220025 had a concentration dependent stimulatory effect on LPS-induced NO production and maximal effect (50%) was achieved at drug concentration of 0,5 μM. The effect of SB220025 was similar to the effect of SB203580 (1 μM) (Fig. 1B). A structurally related control compound SB202474, which does not inhibit p38 MAPK [22], had no effect on NO production. The stimulatory effect of SB220025 was maximal when the compound was added to cells 1 h after LPS (Fig 2A). This result is in line with our previous report in which we showed that the stimulatory effect of SB203580 was maximal when the compound was added 1 h after LPS [19]. The levels of activated p38 peaked in 30 min after LPS, were still high at 1 h and declined gradually thereafter so that activated p38 could be detected even 4 h after LPS (Fig. 2B). Thus, the stimulation of LPS-induced iNOS production by SB220025 could result from inhibition of p38, even when the compound was added to cells 1–2 h after LPS. SB220025 had a clear stimulatory effect also on iNOS protein expression, whereas the negative control compound SB202747 had no effect (Fig. 3A). Interestingly, SB220025 did not increase LPS-induced iNOS mRNA levels when measured 4 h after addition of LPS, whereas a 100% increase in iNOS mRNA levels was observed when measured 10 h after addition of LPS (Fig. 3B).

### SB220025 stabilises iNOS mRNA

Because SB220025 had no effect on iNOS mRNA levels when measured 4 h after LPS, but significantly increased the mRNA levels when measured 10 h after LPS, we hypothesized that SB220025 might stabilize iNOS mRNA.

**Figure 4:**
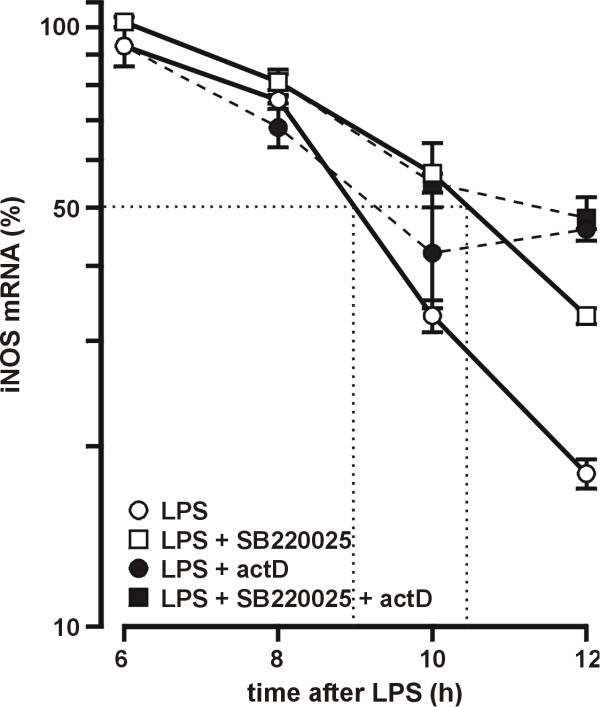
**Effect of SB220025 on iNOS mRNA degradation**. Cells were stimulated with LPS (10 ng/ml) 1 h before addition of SB220025 (0,5 μM). Actinomycin D (actD) (0,5 μg/ml) was added to cells 6 h after LPS. Incubations were terminated at the indicated time points after LPS and total RNA was isolated. iNOS and GAPDH mRNA was measured by real time RT-PCR. iNOS mRNA levels were normalized against GAPDH. Values are mean ± S.E.M. (n = 3).

To study the effect of SB220025 on the stability of iNOS mRNA, the cells were treated with LPS or LPS+SB220025 and cells were incubated for 6 h. Thereafter total RNA was isolated at 2 h intervals. The iNOS mRNA levels in cells were reducing rapidly between 6–12 h after LPS stimulation. The amount of iNOS mRNA in LPS treated cells halved in about 3 h (Fig. 4). The reduction in the amount of iNOS mRNA was slower in cells treated with LPS+SB220025 (iNOS mRNA halved in about 4,5 h).

Actinomycin D (an inhibitor of transcription) was added to cells 6 h after LPS in an attempt to test whether the slowed disappearance of iNOS mRNA in cells treated with LPS+SB220025 was due to increased rate of transcription of iNOS gene or reduced degradation of mRNA. Interestingly, the level of mRNA was reducing at the same or slower rate in cells treated with LPS+actinomycin D compared with cells treated with LPS only, suggesting that no significant transcription of iNOS gene occurs in cells 6 – 12 h after LPS stimulation and that actinomycin D itself inhibits the degradation of iNOS mRNA. Thus, the slowed disappearance of iNOS mRNA in cells treated with SB220025 was most likely due to reduced degradation of mRNA.

### p38α and p38β expression in J774 macrophages

There are four known isoforms of p38 MAPK (α, β, γ and δ) [6], and SB203580 has been shown to inhibit p38α and p38β but not p38γ and p38δ isoforms [23]. p38α and p38β have been recently reported to differently regulate iNOS expression [24]. Therefore we wanted to investigate whether J774.2 macrophages express p38α and p38β isoenzymes.

**Figure 5:**
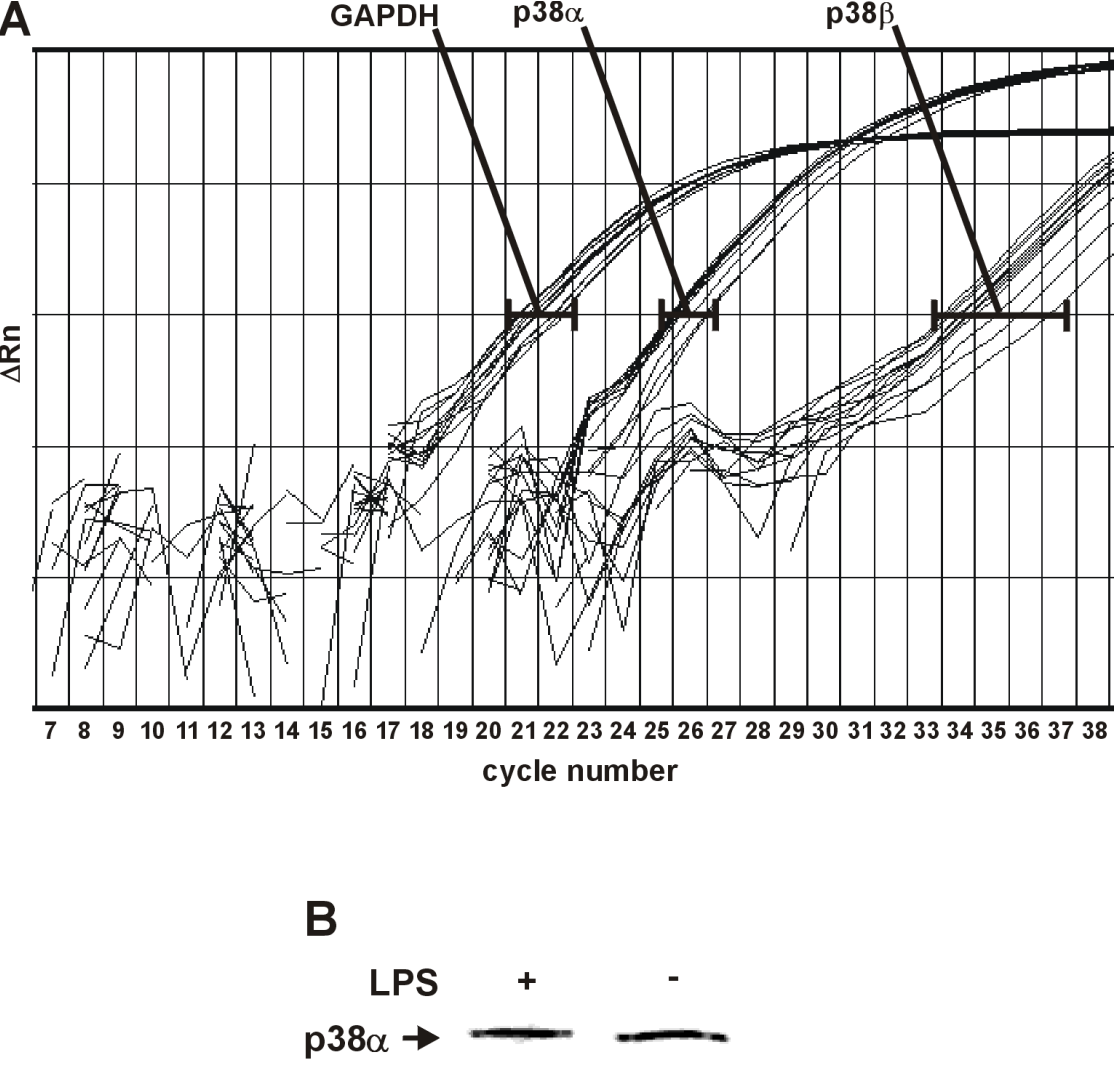
**Expression of p38α and p38β in J774.2 macrophages**. (A) Real time RT-PCR analysis of the p38α, p38β and GAPDH mRNA expression in J774.2 macrophages. Shown is the relationship between the PCR cycle number and the ΔRn which is the normalized reporter signal minus the background noise determined during the PCR cycles. Cells were treated with LPS (10 ng/ml) or vehicle only. Total RNA was isolated after 8 h incubation. Six separate mRNA samples were analysed. (B) Cells were stimulated with LPS. After 24 h, incubations were terminated and immunoblots were run using antibody against p38α. The result is representative of three experiments with similar results.

We used real-time RT-PCR to study the p38α and p38β mRNA expression in J774.2 macrophages. Both unstimulated and LPS stimulated cells expressed p38α mRNA at relatively high level as compared to GAPDH mRNA (Fig. 5A). In contrast, only low level expression of p38β mRNA was detected. In line with the mRNA result, Western blot showed p38α protein expression (Fig. 5B), whereas no p38β protein could be detected by Western blotting.

### SB220025 increases LPS-induced JNK activity

Opposite roles for p38 MAPK and JNK have recently been reported on thrombin induced iNOS expression in RAW264.7 macrophages [25]. JNK and p38 MAPK have common target proteins and there is crosstalk between these signaling cascades [26]. Furthermore, we have previously reported that JNK inhibition destabilizes iNOS mRNA [10]. Therefore we hypothesized that the roles of JNK and p38 MAPK pathways on LPS-induced iNOS expression may be coupled. We continued by investigating whether inhibition of p38 MAPK modulates the activity of JNK.

**Figure 6:**
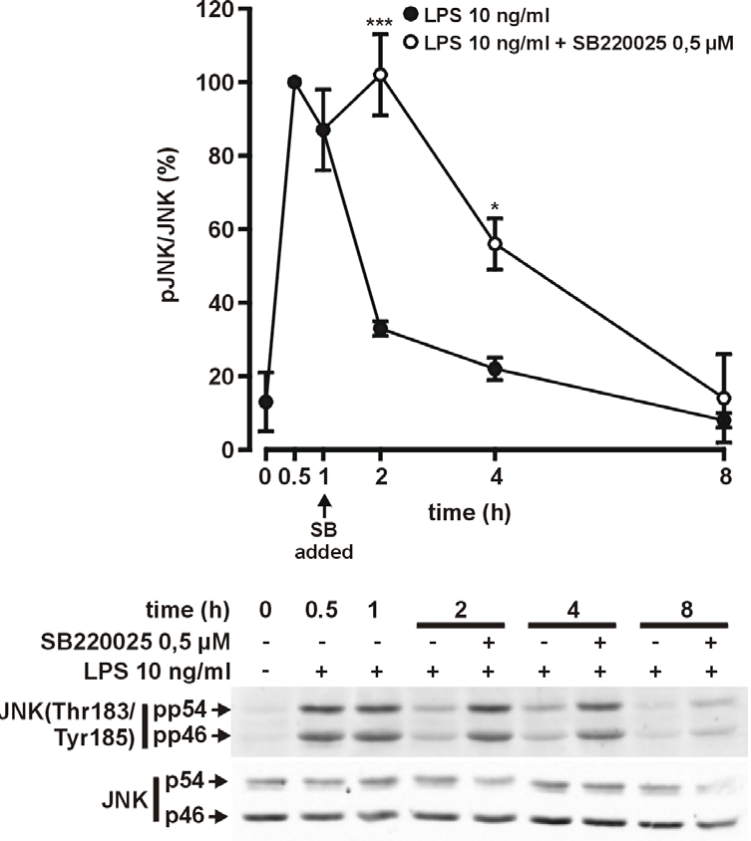
**Effect of SB220025 on JNK activation**. Cells were stimulated with LPS 1 h before addition of SB220025. Incubations were terminated at the indicated time points and parallel immunoblots were run from same cell lysates using antibodies against Thr183/Tyr185 phosphorylated JNK (pp54 and pp46), total JNK (p54 and p46). Phosphorylated JNK values were normalised to total JNK values. Results are expressed as mean ± S.E.M. (n = 3). *P < 0.05, ***P < 0.001 compared with cells treated with LPS only.

**Figure 7:**
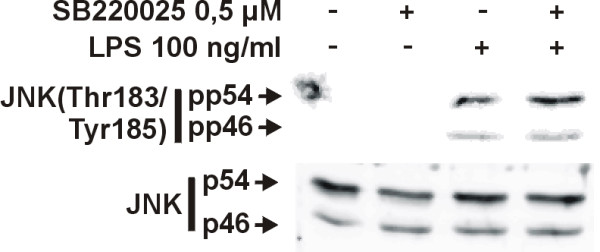
**Effect of SB220025 on JNK activation**. Cells were stimulated with LPS and SB220025. Incubations were terminated after 1 h and parallel immunoblots were run from same cell lysates using antibodies against Thr183/Tyr185 phosphorylated JNK (pp54 and pp46), total JNK (p54 and p46). Phosphorylated JNK values were normalised to total JNK values. The result is representative of three experiments with similar results.

LPS induced a rapid phosphorylation of JNK. The phosphorylation peaked at 0.5 h and declined rapidly thereafter, remaining <33% of the maximum when measured 2–8 h after LPS (Fig. 6). SB220025, when given 1 h after LPS, further increased the LPS-induced JNK phosphorylation compared with cells treated with LPS only. In SB220025-treated cells the amount of phosphorylated JNK remained >55% of the maximum level up to 4 h and declined thereafter. SB220025 alone did not activate JNK (Fig. 7).

**Figure 8:**
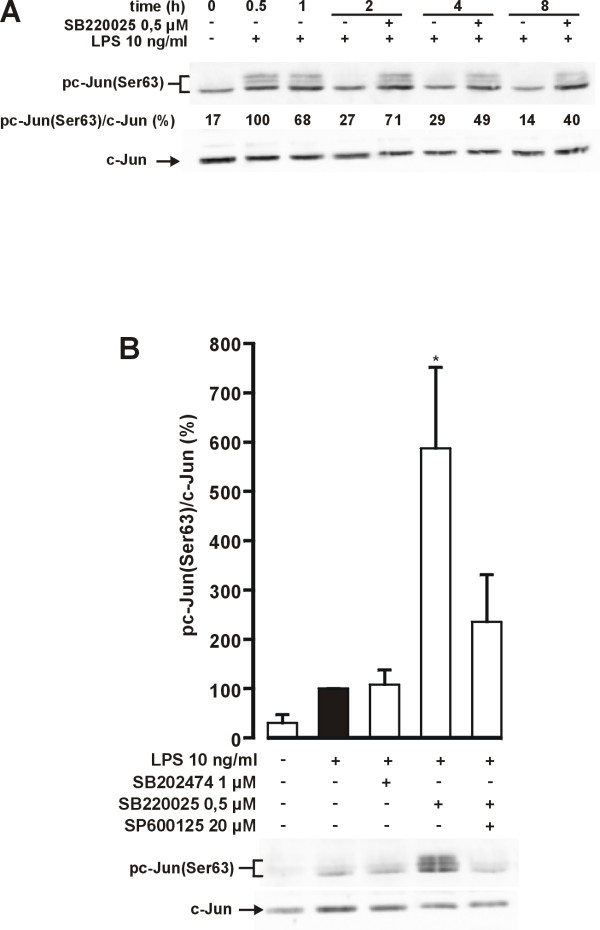
**Effect of SB220025 on JNK activity**. (A) Cells were stimulated with LPS 1 h before addition of SB220025. Incubations were terminated at the indicated time points and parallel immunoblots were run from same cell lysates using antibodies against Ser63 phosphorylated c-Jun (pc-Jun) and total c-Jun. Results are expressed as mean (n = 4). (B) Cells were stimulated with LPS 1 h before addition of tested compounds. Incubations were terminated 2 h after LPS and parallel immunoblots were run from same cell lysates using antibodies against Ser63 phosphorylated c-Jun (pc-Jun) and total c-Jun. Phosphorylated c-Jun values were normalised to total c-Jun values. Results are expressed as mean ± S.E.M (n = 3), *P < 0.05 compared with cells treated with LPS only.

JNK phosphorylates c-Jun at residues Ser63 and Ser73 [27]. In parallel to increased phosphorylation of JNK by SB220025, increased phosphorylation of c-Jun at Ser63 was observed (Fig. 8A). Similar results were obtained when phosphorylation of Ser73 was measured (data not shown). This suggests that the increased phosphorylation of JNK resulted in functionally significant increase in the activity of JNK.

To rule out the possibility, that increased c-Jun phosphorylation was a result of reduced dephosphorylation, we tested whether the effect of SB220025 could be reversed with JNK inhibitor SP600125. Treatment with LPS and SB220025 induced a 6 fold increase in c-Jun Ser63 phosphorylation compared with cells treated with LPS only (Fig. 8B). In contrast, the negative control compound SB202474 had no effect on c-Jun phosphorylation. The SB220025-stimulated increase in c-Jun phosphorylation was almost completely reversed by SP600125, suggesting that the increase in c-Jun phosphorylation was due to increased JNK activity and not due to reduced dephosphorylation.

### The stimulatory effect of SB220025 on LPS-induced NO production and iNOS mRNA expression can be reversed by SP600125

To continue, we hypothesized that the stimulatory effect of SB220025 on LPS-induced NO production was due to increased JNK activity and therefore we tested the effect of JNK inhibitor SP600125 on SB220025-stimulated NO production.

**Figure 9:**
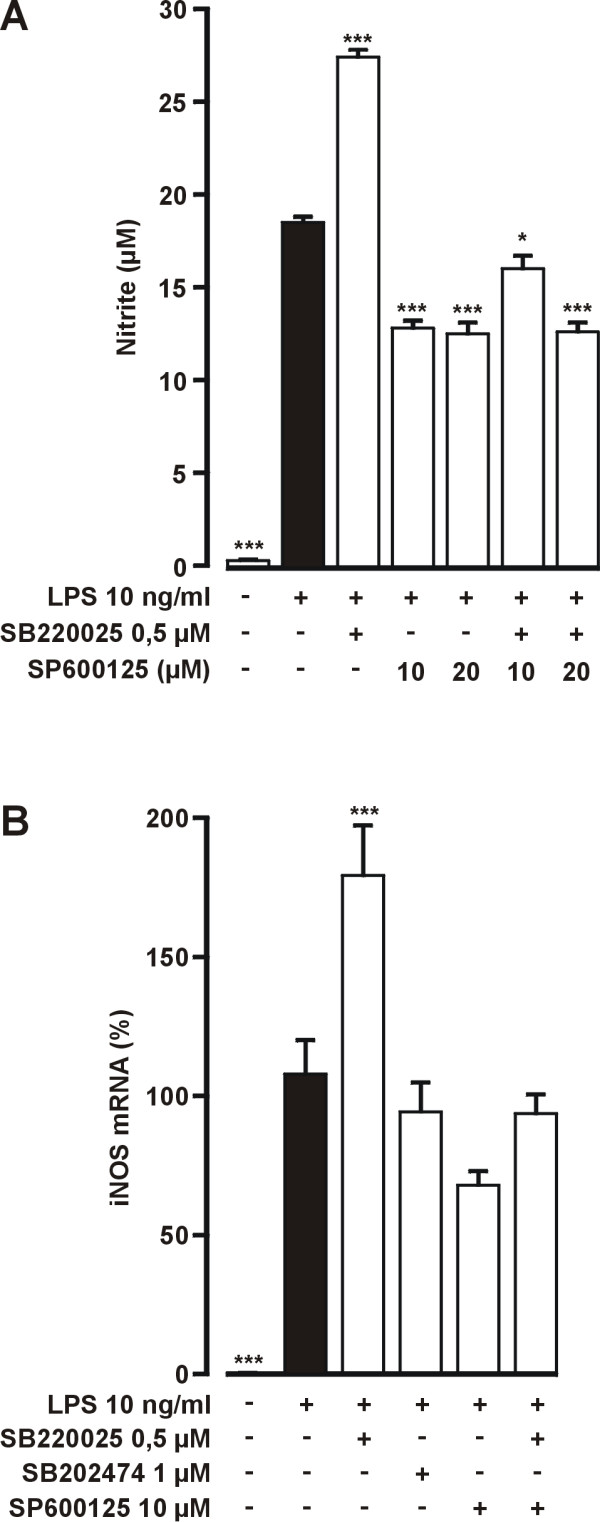
**Effect of SP600125 on LPS and SB220025 stimulated NO production and iNOS mRNA expression**. (A) Cells were stimulated with LPS 1 h before addition of tested compounds. 24 h after addition of LPS the nitrite concentrations were measured as marker of NO production. Values are mean ± S.E.M. (n = 6). (B) Cells were stimulated with LPS 1 h before addition of tested compounds. Incubations were terminated 10 h after LPS and extracted total RNA was subjected to real time RT-PCR. iNOS mRNA levels were normalized against GAPDH. Values are mean ± S.E.M. (n = 4). *P < 0.05, ***P < 0.001 compared with cells treated with LPS only.

SB220025 induced a clear increase in LPS-stimulated NO production, whereas SP600125 inhibited NO production (Fig. 9A). However, when cells were treated with a combination of SB220025 and SP600125 the level of NO production was comparable to levels produced by cells treated with LPS+SP600126. Thus, the effect of SB220025 was reversed by SP600125.

The same result was observed at the level of iNOS mRNA expression. SB220025 increased the amounts of iNOS mRNA to almost two fold compared with cells treated with LPS only, whereas the negative control compound SB202474 had no effect (Fig. 9B). SP600125 alone reduced the LPS-stimulated iNOS mRNA levels slightly. In addition, in the presence of the JNK inhibitor SP600125, SB220025 had no stimulatory effect on iNOS mRNA levels.

### Cycloheximide increases JNK activity and iNOS mRNA expression

**Figure 10:**
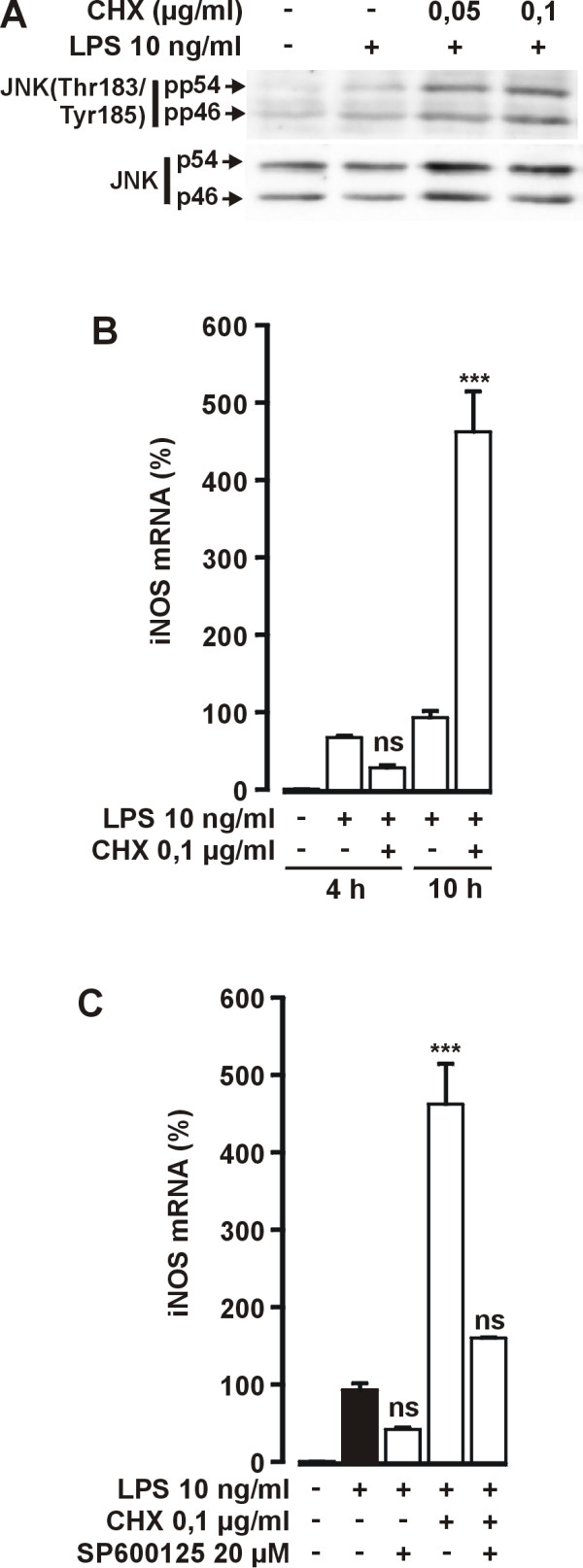
**Effect of cycloheximide (CHX) on LPS-induced JNK activation and iNOS mRNA expression**. (A) Cells were stimulated with LPS 1 h before addition of different concentrations of CHX. Incubations were terminated 2 h after LPS and parallel immunoblots were run from same cell lysates using antibodies against Thr183/Tyr185 phosphorylated JNK (pp54 and pp46), total JNK (p54 and p46). The result is representative of two experiments with similar results. (B) Cells were stimulated with LPS 1 h before addition of CHX. Incubations were terminated at indicated time points and extracted total RNA was subjected to real time RT-PCR. iNOS mRNA levels were normalised against GAPDH. Values are mean ± S.E.M. (n = 3). (C) Cells were stimulated with LPS 1 h before addition of tested compounds. Incubations were terminated 10 h after LPS and extracted total RNA was subjected to real time RT-PCR. iNOS mRNA levels were normalized against GAPDH. Values are mean ± S.E.M. (n = 3). ***P < 0.001.

Cycloheximide is widely used as an inhibitor of protein synthesis. However, cycloheximide also activates JNK [28]. Therefore we continued by investigating whether cycloheximide has similar effect on iNOS mRNA expression as SB220025. Cycloheximide at 0,05–0,1 μg/ml concentrations increased LPS-induced JNK activity (Fig. 10A). Interestingly, cycloheximide had no significant effect on iNOS mRNA expression when measured 4 h after LPS, but increased iNOS mRNA levels >4 fold when measured 10 h after LPS (Fig. 10B). Furthermore, the effect of cycloheximide on iNOS mRNA expression was partially inhibited by SP600125 (Fig. 10C). These results show that the effect of cycloheximide on JNK activity and iNOS expression were very similar to the effect of SB220025.

## Discussion

In the present study we have shown that inhibition of p38 MAPK by SB220025 increases LPS-induced JNK activity, which leads to stabilization of iNOS mRNA and increased iNOS expression and NO production in J774.2 macrophages.

Inhibitors of p38 MAPK have been shown to up-regulate iNOS expression in IL-1β-stimulated rat mesangial cells [14,24], in LPS+IFN-γ-stimulated RAW264.7γ macrophages [15], in interferon-γ (IFN-γ)+mannose-capped lipoarabinomannan-stimulated RAW264.7γ macrophages [16] and in LPS-stimulated J774.A1 macrophages [19]. In this study, a novel p38 MAPK inhibitor SB220025 increased LPS-induced NO production with an EC_50 _of ~100 nM, which is close to its IC_50 _value of p38 MAPK inhibition (~60 nM) [21]. Furthermore, a structurally related inactive control compound SB202474 had no effect. These results together suggest that the observed increase in NO production and iNOS expression was due to inhibition of p38 MAPK. SB203580 inhibits the p38α and p38β isoforms at equipotent efficiency, but does not inhibit p38γ or p38δ [23]. To our knowledge there is no published data about the isoform specificity of SB220025. In the present study both SB203580 and SB220025 had similar effect on LPS-induced NO production, thus it is likely that the observed effects are mediated by p38α and/or p38β. J774 macrophages were found to express p38α mRNA and p38α protein at relatively high levels whereas only low amounts of p38β mRNA were detected. Similar pattern of p38α and p38β expression was reported by Lui et al. (2004) in rat renal mesangial cells, in which p38 MAPK inhibition was also found to increase iNOS expression. In their further transfection experiments, Lui et al. (2004) found that p38α mutant and p38β wild-type isoforms inhibited IL-1-induced iNOS expression suggesting that the two isoforms have reciprocal effects on iNOS expression in renal mesangial cells. Our results show that inhibition of p38 enhances iNOS expression and NO production in macrophages activated by LPS but further studies are required to clarify the roles of different p38 MAPK isoforms in that process.

The mechanisms how p38 MAPK inhibitors enhance iNOS expression and NO production have been unclear. The present data suggest that inhibition of p38 enhances JNK activity that results in stabilization of iNOS mRNA and enhanced iNOS protein expression. Our results are in line with those of Avdi et al. (2002) in which inhibition of p38 MAPK by SB203580 led to increased activity of JNK in human neutrophils [29]. The inhibition of p38 MAPK was found to reduce the activity of protein phosphatase-2A which resulted in reduced dephosphorylation and increased activity of JNK. Various protein phosphatases are able to dephosphorylate MAPKs and are thus important regulators of MAPK activity [30]. It is possible that p38 MAPK regulates the activity of protein phosphatase-2A or some other protein phosphatase and inhibition of p38 MAPK by SB220025 reduces protein phosphatase activity, which leads to increased JNK activity observed in the present study. Interestingly, we found that JNK inhibitor SP600125 reversed the SB220025 stimulated increase in JNK activity, NO production and iNOS expression, suggesting that increased iNOS expression by SB220025 results from increased JNK activity. In addition, cycloheximide, a known JNK activator, also increased LPS-induced iNOS mRNA expression in a similar manner as SB220025. The stimulatory effect of cycloheximide on iNOS mRNA expression was reversed by SP600125, suggesting that the effect of cycloheximide is at least partially mediated through increased JNK activity. Up-regulatory role for JNK in iNOS expression has been previously shown in IL-1+IFN-γ-stimulated human fetal astrocytes [9], in LPS+IFN-γ-stimulated RAW264.7γ macrophages [15], IL-1β-stimulated rat primary mesangial cells [8]and LPS-stimulated J774.A1 macrophages [10].

Regulation of iNOS mRNA stability seems to be an important mean to regulate iNOS expression. However, the mechanisms regulating iNOS mRNA stability are poorly known. HuR is a mRNA stabilizing factor, which has been shown to bind an AU-rich sequence element in the 3' untranslated region of human iNOS mRNA and to stabilise iNOS mRNA [31]. Tristetraprolin seems to have a role as a mRNA stabilizing factor for human iNOS [32] while the KH-type splicing regulatory protein (KSRP) has been identified as a destabilizing factor [33]. Heterogeneous nuclear ribonucleoproteins I and L have been reported to interact with murine iNOS mRNA [34]. In addition, dexamethasone [35], protein kinase Cδ [36] and β-adrenergic stimulation [37] have been shown to regulate iNOS mRNA stability. Recently, we have shown that JNK inhibitor SP600125 reduces iNOS mRNA stability [10]. In the present study, treatment with SB220025 had no effect on iNOS mRNA levels when measured 4 h after LPS stimulation, whereas a two fold increase in mRNA levels was observed 10 h after LPS. Furthermore, mRNA levels decreased slower in SB220025 treated cells than in cells treated with LPS alone. These results together suggest that SB220025 increases iNOS mRNA expression by stabilising mRNA. Also actinomycin D seems to have a stabilising effect on iNOS mRNA. Actinomycin D has previously been reported to stabilise mRNAs of transferrin receptor [38] and cyclooxygenase-2 [39] but the mechanisms are not known in detail.

## Conclusion

The present results show that inhibition of p38 MAPK enhances JNK activity, which leads to stabilisation of iNOS mRNA, and to increased iNOS expression and NO production. p38 MAPK regulates activity of JNK pathway and it is therefore important to consider whether results obtained by inhibiting p38 MAPK might result from increased JNK activity rather than from reduced p38 MAPK activity directly. Finally, based on our results, it seems that JNK is an important post-transcriptional regulator of LPS-induced iNOS expression and NO production.

## Methods

### Materials

Reagents were obtained as follows: anthra(1,9-cd)pyrazol-6(2H)-one (SP600125), 4-ethyl-2-(4-methoxyphenyl)-5-(4-hydroxyphenyl)-5-(4pyridyl)-imidazole (SB202474), 5-(2-amino-4-pyrimidinyl)-4-(4-fluorophenyl)-1-(4-piperidinyl)-imidazole(SB220025) and 4-(4-fluorophenyl)-2-(4-methylsulfinylphenyl)-5-(4-pyridyl)-imidazole (SB203580) (Calbiochem), rabbit polyclonal mouse iNOS, c-Jun, JNK1 and actin antibodies, goat polyclonal p38β antibody, donkey anti-goat antibody and goat anti-rabbit polyclonal antibody (Santa Cruz Biotechnology Inc.), rabbit polyclonal p38 MAPK, phospho-p38 MAPK (Thr180/Tyr182), p38α, phospho-SAPK/JNK (Thr183/Tyr185) and phospho-c-Jun (Ser63) II antibodies (Cell Signaling technology). All other reagents were from Sigma.

### Cell culture

J774.2 macrophages (The European Collection of Cell Cultures) were cultured at 37°C, 5% CO_2 _atmosphere, in Dulbecco's Modified Eagle's Medium with glutamax-I (Cambrex Bioproducts) containing 10% of heat inactivated foetal bovine serum, 100 U/ml penicillin, 100 μg/ml streptomycin and 250 ng/ml amphotericin B (all from Invitrogen). Cells were seeded on 24 well plates for nitrite measurements and in 6 well plates for Western blot and RT-PCR and grown for 48 h prior to experiments.

### Nitrite assays

At indicated time points the culture medium was collected for nitrite measurement, which was used as a measure of NO production. Culture medium (100 μl) was incubated with 100 μl of Griess reagent (0.1% napthalethylenediamine dihydrochloride, 1% sulphanilamine, 2.5% H_3_PO_4_) and the absorbance was measured at 540 nm.

### Preparation of cell lysates

At indicated time points cells were rapidly washed with ice cold PBS and solubilised in cold lysis buffer containing 10 mM Tris-base, 5 mM EDTA, 50 mM NaCl, 1% Triton-X-100, 0.5 mM phenylmethylsulfonyl fluoride, 2 mM sodiumorthovanadate, 10 μg/ml leupeptin, 25 μg/ml aprotinin, 1.25 mM NaF, 1 mM sodiumpyrophosphate and 10 mM n-octyl-β-D-glucopyranoside. After incubation for 20 min on ice, lysates were centrifuged (14500 g, 15 min) and supernatants were mixed 1:4 with SDS loading buffer (62.5 mM Tris-HCl, pH 6.8, 10% glycerol, 2% SDS, 0.025% bromophenol blue, 5% β-mercaptoethanol) and boiled for 5 min. Protein concentrations in the samples were measured by the Coomassie blue method [40].

### Western blotting

Protein (30 μg) was loaded on 10% SDS-polyacrylamide electrophoresis gel and electrophoresed for 4 h at 100 V in buffer containing 95 mM Tris-HCl, 960 mM glycine and 0.5% SDS. After electrophoresis the proteins were transferred to Hybond ECL™ nitrocellulose membrane (Amersham) with semi-dry blotter at 2.5 mA/cm^2 ^for 60 min. After transfer the membrane was blocked in TBS/T (20 mM Tris-base pH 7.6, 150 mM NaCl, 0.1% Tween-20) containing 5% bovine serum albumin for 1 h at room temperature and incubated with primary antibody in the blocking solution at 4°C overnight. Thereafter the membrane was washed 4× with TBS/T for 5 min, incubated with secondary antibody in the blocking solution for 0.5 h at room temperature and washed 4× with TBS/T for 5 min. Bound antibody was detected using SuperSignal^® ^West Pico chemiluminescent substrate (Pierce) and FluorChem™ 8800 imaging system (Alpha Innotech). The quantitation of chemiluminescent signal was carried out with FluorChem™ software v. 3.1.

### RNA extraction and real-time RT-PCR

At indicated time points cell monolayers were rapidly washed with ice cold PBS and cells were homogenised using QIAshredder™ (QIAGEN Inc.). RNA extraction was carried out with RNeasy^® ^kit for isolation of total RNA (QIAGEN inc.). Total RNA (25 ng) was reverse transcribed to cDNA using TaqMan Reverse Transcription reagents and random hexamers (Applied Biosystems). Reverse transcriptase (RT) reaction parameters were as follows: incubation at 25°C for 10 min, RT at 48°C for 30 min and RT inactivation at 95°C for 5 min. cDNA obtained from the RT reaction (amount corresponding approximately 1 ng of total RNA) was subjected to PCR using TaqMan^® ^Universal PCR Master Mix and ABI PRISM^® ^7000 Sequence detection system (Applied Biosystems). GAPDH and iNOS primer and probe sequences and concentrations were optimised according to manufacturers guidelines in TaqMan^® ^Universal PCR Master Mix Protocol Part Number 4304449 Rev. C and were as follows: 5'-CCTGGTACGGGCATTGCT-3', 5'-GCTCATGCGGCCTCCTT-3' (forward and reverse mouse iNOS primer respectively, both 300 nM), 5'-CAGCAGCGGCTCCATGACTCCC-3'(mouse iNOS probe 150 nM, containing 6-FAM as 5'-reporter dye) and 5'-GCATGGCCTTCCGTGTTC-3', 5'-GATGTCATCATACTTGGCAGGTTT-3' (forward and reverse mouse glyceraldehyde-3-phosphate dehydrogenase (GAPDH) primer respectively, both 300 nM), 5'-TCGTGGATCTGACGTGCCGCC-3'(mouse GAPDH probe 150 nM, containing 6-FAM as 5'-reporter dye) (Metabion). Primers and probes for p38α (product Mm00442497_m1) and p38β (product Mm00440955_m1) (Applied Biosystems) were used as recommended by the manufacturer. PCR reaction parameters were as follows: incubation at 50°C for 2 min, incubation at 95°C for 10 min and thereafter 40 cycles of denaturation at 95°C for 15 sec and annealing and extension at 60°C for 1 min. Each sample was determined in duplicate.

A standard curve method was used to determine the relative iNOS and GAPDH mRNA levels as described in Applied Biosystems User Bulletin #2. In short, a standard curve for each gene was created using mRNA isolated from LPS-stimulated J774.2 macrophages. Isolated RNA was reverse transcribed as described. Dilution series were made from obtained cDNA ranging from 10 ng to 1 pg and were subjected to real time PCR as described. Obtained threshold cycle values were plotted against dilution factor to create a standard curve. Relative mRNA levels in test samples were then calculated from the standard curve.

### Statistics

Results are expressed as mean ± standard error of mean (S.E.M.). When indicated, statistical significance was calculated by analysis of variances supported by Bonferroni multiple comparisons test. Differences were considered significant at *P *< 0.05.

## Authors' contributions

AL participated in the design of the study, carried out most of the laboratory work and drafted the manuscript. OS did laboratory work and helped to draft the manuscript. HK participated in the design of the study and in the writing of the manuscript. EM participated in the design of the study and in the writing of the manuscript.

## References

[B1] Moilanen E, Whittle B, Moncada S, Gallin JI, Snyderman R (1999). Nitric oxide as a factor in inflammation. Inflammation: Basic principles and clinical correlates.

[B2] Korhonen R, Lahti A, Kankaanranta H, Moilanen E (2005). Nitric oxide production and signaling in inflammation. Curr Drug Targets Inflamm Allergy.

[B3] Alderton WK, Cooper CE, Knowles RG (2001). Nitric oxide synthases: structure, function and inhibition. Biochem J.

[B4] Vallance P, Leiper J (2002). Blocking NO synthesis: how, where and why?. Nat Rev Drug Discov.

[B5] Kleinert H, Schwarz PM, Forstermann U (2003). Regulation of the expression of inducible nitric oxide synthase. Biol Chem.

[B6] Chang L, Karin M (2001). Mammalian MAP kinase signalling cascades. Nature.

[B7] Dong C, Davis RJ, Flavell RA (2002). MAP kinases in the immune response. Annu Rev Immunol.

[B8] Guan Z, Buckman SY, Springer LD, Morrison AR (1999). Both p38alpha(MAPK) and JNK/SAPK pathways are important for induction of nitric-oxide synthase by interleukin-1beta in rat glomerular mesangial cells. J Biol Chem.

[B9] Hua LL, Zhao ML, Cosenza M, Kim MO, Huang H, Tanowitz HB, Brosnan CF, Lee SC (2002). Role of mitogen-activated protein kinases in inducible nitric oxide synthase and TNFalpha expression in human fetal astrocytes. J Neuroimmunol.

[B10] Lahti A, Jalonen U, Kankaanranta H, Moilanen E (2003). c-Jun NH2-terminal kinase inhibitor anthra(1,9-cd)pyrazol-6(2H)-one reduces inducible nitric-oxide synthase expression by destabilizing mRNA in activated macrophages. Mol Pharmacol.

[B11] Chen C, Chen YH, Lin WW (1999). Involvement of p38 mitogen-activated protein kinase in lipopolysaccharide-induced iNOS and COX-2 expression in J774 macrophages. Immunology.

[B12] Chen CC, Wang JK (1999). p38 but not p44/42 mitogen-activated protein kinase is required for nitric oxide synthase induction mediated by lipopolysaccharide in RAW 264.7 macrophages [published erratum appears in Mol Pharmacol 1999 Jun;55(6):1108]. Mol Pharmacol.

[B13] Bhat NR, Feinstein DL, Shen Q, Bhat AN (2002). p38 MAPK-mediated transcriptional activation of inducible nitric-oxide synthase in glial cells. Roles of nuclear factors, nuclear factor kappa B, cAMP response element-binding protein, CCAAT/enhancer-binding protein-beta, and activating transcription factor-2. J Biol Chem.

[B14] Guan Z, Baier LD, Morrison AR (1997). p38 mitogen-activated protein kinase down-regulates nitric oxide and up-regulates prostaglandin E2 biosynthesis stimulated by interleukin-1beta. J Biol Chem.

[B15] Chan ED, Riches DW (2001). IFN-gamma + LPS induction of iNOS is modulated by ERK, JNK/SAPK, and p38(mapk) in a mouse macrophage cell line. Am J Physiol Cell Physiol.

[B16] Chan ED, Morris KR, Belisle JT, Hill P, Remigio LK, Brennan PJ, Riches DW (2001). Induction of inducible nitric oxide synthase-NO* by lipoarabinomannan of Mycobacterium tuberculosis is mediated by MEK1-ERK, MKK7-JNK, and NF-kappaB signaling pathways. Infect Immun.

[B17] Chan ED, Winston BW, Uh ST, Wynes MW, Rose DM, Riches DW (1999). Evaluation of the role of mitogen-activated protein kinases in the expression of inducible nitric oxide synthase by IFN-gamma and TNF-alpha in mouse macrophages. J Immunol.

[B18] Cho MK, Suh SH, Kim SG (2002). JunB/AP-1 and NF-kappa B-mediated induction of nitric oxide synthase by bovine type I collagen in serum-stimulated murine macrophages. Nitric Oxide.

[B19] Lahti A, Kankaanranta H, Moilanen E (2002). P38 mitogen-activated protein kinase inhibitor SB203580 has a bi-directional effect on iNOS expression and NO production. Eur J Pharmacol.

[B20] Cuenda A, Rouse J, Doza YN, Meier R, Cohen P, Gallagher TF, Young PR, Lee JC (1995). SB 203580 is a specific inhibitor of a MAP kinase homologue which is stimulated by cellular stresses and interleukin-1. FEBS Lett.

[B21] Jackson JR, Bolognese B, Hillegass L, Kassis S, Adams J, Griswold DE, Winkler JD (0025). Pharmacological effects of SB 22 a selective inhibitor of P38 mitogen-activated protein kinase, in angiogenesis and chronic inflammatory disease models. J Pharmacol Exp Ther.

[B22] Lee JC, Laydon JT, McDonnell PC, Gallagher TF, Kumar S, Green D, McNulty D, Blumenthal MJ, Heys JR, Landvatter SW (1994). A protein kinase involved in the regulation of inflammatory cytokine biosynthesis. Nature.

[B23] Kumar S, McDonnell PC, Gum RJ, Hand AT, Lee JC, Young PR (1997). Novel homologues of CSBP/p38 MAP kinase: activation, substrate specificity and sensitivity to inhibition by pyridinyl imidazoles. Biochem Biophys Res Commun.

[B24] Lui P, Zeng C, Acton S, Cok S, Sexton A, Morrison AR (2004). Effects of p38MAPK isoforms on renal mesangial cell inducible nitric oxide synthase expression. Am J Physiol Cell Physiol.

[B25] Kang KW, Choi SY, Cho MK, Lee CH, Kim SG (2003). Thrombin induces nitric-oxide synthase via Galpha12/13-coupled protein kinase C-dependent I-kappaBalpha phosphorylation and JNK-mediated I-kappaBalpha degradation. J Biol Chem.

[B26] Hommes DW, Peppelenbosch MP, van Deventer SJ (2003). Mitogen activated protein (MAP) kinase signal transduction pathways and novel anti-inflammatory targets. Gut.

[B27] Derijard B, Hibi M, Wu IH, Barrett T, Su B, Deng T, Karin M, Davis RJ (1994). JNK1: a protein kinase stimulated by UV light and Ha-Ras that binds and phosphorylates the c-Jun activation domain. Cell.

[B28] Newton R, Stevens DA, Hart LA, Lindsay M, Adcock IM, Barnes PJ (1997). Superinduction of COX-2 mRNA by cycloheximide and interleukin-1beta involves increased transcription and correlates with increased NF-kappaB and JNK activation. FEBS Lett.

[B29] Avdi NJ, Malcolm KC, Nick JA, Worthen GS (2002). A role for protein phosphatase-2A in p38 mitogen-activated protein kinase-mediated regulation of the c-Jun NH(2)-terminal kinase pathway in human neutrophils. J Biol Chem.

[B30] Keyse SM (2000). Protein phosphatases and the regulation of mitogen-activated protein kinase signalling. Curr Opin Cell Biol.

[B31] Rodriguez-Pascual F, Hausding M, Ihrig-Biedert I, Furneaux H, Levy AP, Forstermann U, Kleinert H (2000). Complex contribution of the 3'-untranslated region to the expressional regulation of the human inducible nitric-oxide synthase gene. Involvement of the RNA-binding protein HuR. J Biol Chem.

[B32] Fechir M, Linker K, Pautz A, Hubrich T, Forstermann U, Rodriguez-Pascual F, Kleinert H (2005). Tristetraprolin regulates the expression of the human inducible nitric-oxide synthase gene. Mol Pharmacol.

[B33] Linker K, Pautz A, Fechir M, Hubrich T, Greeve J, Kleinert H (2005). Involvement of KSRP in the post-transcriptional regulation of human iNOS expression-complex interplay of KSRP with TTP and HuR. Nucleic Acids Res.

[B34] Soderberg M, Raffalli-Mathieu F, Lang MA (2002). Inflammation modulates the interaction of heterogeneous nuclear ribonucleoprotein (hnRNP) I/polypyrimidine tract binding protein and hnRNP L with the 3'untranslated region of the murine inducible nitric-oxide synthase mRNA. Mol Pharmacol.

[B35] Korhonen R, Lahti A, Hamalainen M, Kankaanranta H, Moilanen E (2002). Dexamethasone inhibits inducible nitric-oxide synthase expression and nitric oxide production by destabilizing mRNA in lipopolysaccharide-treated macrophages. Mol Pharmacol.

[B36] Carpenter L, Cordery D, Biden TJ (2001). Protein kinase Cdelta activation by interleukin-1beta stabilizes inducible nitric-oxide synthase mRNA in pancreatic beta-cells. J Biol Chem.

[B37] Gustafsson AB, Brunton LL (2000). beta-adrenergic stimulation of rat cardiac fibroblasts enhances induction of nitric-oxide synthase by interleukin-1beta via message stabilization. Mol Pharmacol.

[B38] Seiser C, Posch M, Thompson N, Kuhn LC (1995). Effect of transcription inhibitors on the iron-dependent degradation of transferrin receptor mRNA. J Biol Chem.

[B39] Dixon DA, Kaplan CD, McIntyre TM, Zimmerman GA, Prescott SM (2000). Post-transcriptional control of cyclooxygenase-2 gene expression. The role of the 3'-untranslated region. J Biol Chem.

[B40] Bradford MM (1976). A rapid and sensitive method for the quantitation of microgram quantities of protein utilizing the principle of protein-dye binding. Anal Biochem.

